# Higher Mortality Rate in Moderate-to-Severe Thoracoabdominal Injury Patients with Admission Hyperglycemia Than Nondiabetic Normoglycemic Patients

**DOI:** 10.3390/ijerph16193562

**Published:** 2019-09-25

**Authors:** Wei-Ti Su, Shao-Chun Wu, Sheng-En Chou, Chun-Ying Huang, Shiun-Yuan Hsu, Hang-Tsung Liu, Ching-Hua Hsieh

**Affiliations:** 1 Department of Trauma Surgery, Kaohsiung Chang Gung Memorial Hospital, Chang Gung University and College of Medicine, Kaohsiung 83301, Taiwan; s101132@adm.cgmh.org.tw (W.-T.S.); athenechou@gmail.com (S.-E.C.); junyinhaung@yahoo.com.tw (C.-Y.H.); ah.lucy@hotmail.com (S.-Y.H.); 2 Department of Anesthesiology, Kaohsiung Chang Gung Memorial Hospital, Chang Gung University and College of Medicine, Kaohsiung 83301, Taiwan; shaochunwu@gmail.com; 3 Department of Plastic Surgery, Kaohsiung Chang Gung Memorial Hospital, Chang Gung University and College of Medicine, Kaohsiung 83301, Taiwan

**Keywords:** admission hyperglycemia, stress-induced hyperglycemia, diabetic hyperglycemia, diabetes mellitus, thoracoabdominal trauma, mortality

## Abstract

Background: Hyperglycemia at admission is associated with an increase in worse outcomes in trauma patients. However, admission hyperglycemia is not only due to diabetic hyperglycemia (DH), but also stress-induced hyperglycemia (SIH). This study was designed to evaluate the mortality rates between adult moderate-to-severe thoracoabdominal injury patients with admission hyperglycemia as DH or SIH and in patients with nondiabetic normoglycemia (NDN) at a level 1 trauma center. Methods: Patients with a glucose level ≥200 mg/dL upon arrival at the hospital emergency department were diagnosed with admission hyperglycemia. Diabetes mellitus (DM) was diagnosed when patients had an admission glycohemoglobin A1c ≥6.5% or had a past history of DM. Admission hyperglycemia related to DH and SIH was diagnosed in patients with and without DM. Patients who had a thoracoabdominal Abbreviated Injury Scale score <3, a polytrauma, a burn injury and were below 20 years of age were excluded. A total of 52 patients with SIH, 79 patients with DH, and 621 patients with NDN were included from the registered trauma database between 1 January 2009, and 31 December 2018. To reduce the confounding effects of sex, age, comorbidities, and injury severity of patients in assessing the mortality rate, different 1:1 propensity score-matched patient populations were established to assess the impact of admission hyperglycemia (SIH or DH) vs. NDN, as well as SIH vs. DH, on the outcomes. Results: DH was significantly more frequent in older patients (61.4 ± 13.7 vs. 49.8 ± 17.2 years, *p* < 0.001) and in patients with higher incidences of preexisting hypertension (2.5% vs. 0.3%, *p* < 0.001) and congestive heart failure (3.8% vs. 1.9%, *p* = 0.014) than NDN. On the contrary, SIH had a higher injury severity score (median [Q1–Q3], 20 [15–22] vs. 13 [10–18], *p* < 0.001) than DH. In matched patient populations, patients with either SIH or DH had a significantly higher mortality rate than NDN patients (10.6% vs. 0.0%, *p* = 0.022, and 5.3% vs. 0.0%, *p* = 0.043, respectively). However, the mortality rate was insignificantly different between SIH and DH (11.4% vs. 8.6%, odds ratio, 1.4; 95% confidence interval, 0.29–6.66; *p* = 0.690). Conclusion: This study revealed that admission hyperglycemia in the patients with thoracoabdominal injuries had a higher mortality rate than NDN patients with or without adjusting the differences in patient’s age, sex, comorbidities, and injury severity.

## 1. Background

Thoracoabdominal injury is one of the leading causes of mortality in trauma patients. In polytrauma patients, abdominal injury and thoracic trauma are the second and third most common causes of death, respectively, after head trauma [1]. Approximately 60% of cases are related to traffic accidents [2,3], followed by acts of violence and fall accidents [4]. During a side-impact car crash, a multivariate analysis of 68,124 occupants revealed that advanced age and no use of seat belt were significantly associated with a major injury to the thoracoabdominal region [5]. Additionally, the proximity of the seat to the side of the crash was related to the severity of the thoracoabdominal injury [6]. Furthermore, a blow on the lower chest wall also places the upper abdominal organs at risk of injury. Most commonly, the impact to the lower chest wall can result in spleen or liver injures [7,8]. Conversely, an impact on the abdomen leads to intrathoracic injury, such as pneumothorax [9]. In a review of 1661 patients with blunt thoracoabdominal injuries, the overall incidences of hollow viscus injuries and intra-abdominal solid organ injuries were 6.0% and 59.7%, respectively [10]. The overall mortality rate varied from 5.1% to 9.3% [2,11,12].

It is well known that hyperglycemia and insulin resistance are often accompanied with severe illness [13]. In 2003, a retrospective study of trauma patients demonstrated that an admission hyperglycemia with glucose levels greater than 200 mg/dL was independently associated with an increased rate of mortality [14]. Additionally, admission hyperglycemia is associated with increased mortality rates both in critically ill [15,16,17] and noncritically ill trauma patients [14,18]. However, admission hyperglycemia is not only attributed to diabetic hyperglycemia (DH) but also stress-induced hyperglycemia (SIH). SIH is a hyperglycemia caused by a stress response and characterized by aggravated gluconeogenesis and glycogenolysis, with up to ten times greater cortical output of the adrenal function [19]. The excessive counter-regulatory hormone leads to relative insulin deficiency and temporary insulin resistance, followed by an uncompensated hyperglycemia [20] and a failure to suppress hepatic gluconeogenesis [21]. SIH frequently occurs in trauma patients [18,22,23] and is associated with worse outcomes, like an increased rate of postoperative wound infection, longer stay in intensive care unit, and increased mortality [18,24,25,26,27,28]. Kerby et al. had reported that in patients with all trauma causes, SIH was associated with a higher rate of mortality than DH [29]. We have also reported that in trauma patients [30] or in patients with traumatic brain injury [31], a worse mortality outcome was only observed in patients with SIH, but not DH, when adjusting the baseline difference of age, sex, preexisting comorbidities, and injury severity. Because the pathophysiological response of patients with thoracoabdominal injuries is different from those who had a traumatic brain injury, this study was designed to compare the mortality rates between the thoracoabdominal injury patients with SIH or DH and patients with nondiabetic normoglycemia (NDN). The primary outcome was the mortality rate of these patients.

## 2. Methods

### 2.1. Ethics Statement

After obtaining the approval (referencing number: 201900726B0) from the institutional review board (IRB) of Chang Gung Memorial Hospital, a level 1 trauma center located in Southern Taiwan [32,33,34], we retrospectively reviewed the hospitalized patients with thoracoabdominal injuries from 1 January 2009, to 31 December 2018. Because of the retrospective study design, informed consent of the patients was waived in the study according to the regulations of the IRB.

### 2.2. Study Population

**Figure 1:**
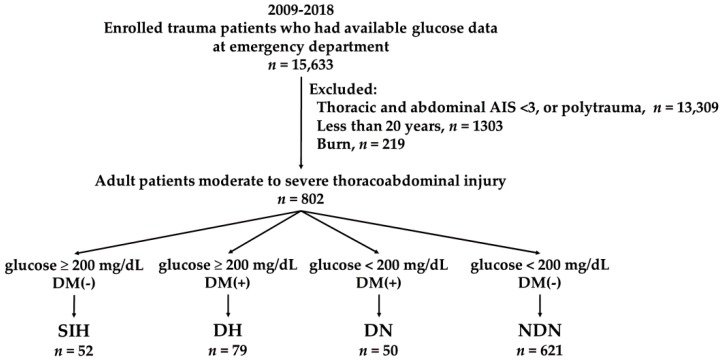
A flowchart in grouping the adult patients with thoracoabdominal injuries into the following subgroups: stress-induced hyperglycemia (SIH), diabetic hyperglycemia (DH), diabetic normoglycemia (DN), and nondiabetic normoglycemia (NDN). DM, diabetes mellitus.

Admission hyperglycemia was defined as patients with a glucose level ≥200 mg/dL in the serum upon arrival at the emergency department (ED). According to the guidelines of the American Diabetes Association, diabetes mellitus (DM) was diagnosed [35] when patients had a past history of DM or had an admission glycohemoglobin A1c ≥ 6.5%. We only included those patients who had available glucose data at the ED (*n* = 15,633) in this study (Figure 1). Moderate-to-severe thoracoabdominal injury was defined as the presence of a thoracic or abdominal Abbreviated Injury Scale (AIS) score ≥ 3. In this study, we excluded patients with a thoracoabdominal AIS < 3; those who had polytrauma, which was defined as if there were any additional AIS scores ≥ 3 in other regions of the body [36] (*n* = 13,309); those who were less than 20 years old (*n* = 1303); and those who had a burn injury (*n* = 219). Accordingly, these adult patients with moderate-to-severe thoracoabdominal injuries (*n* = 802) were grouped into the following four exclusive subgroups: (1) SIH (admission hyperglycemia in patients without DM) (*n* = 52), (2) DH (admission hyperglycemia in patients with DM) (*n* = 79), (3) DN (no admission hyperglycemia in patients with DM) (*n* = 50), and (4) NDN (the patients with no admission hyperglycemia and without DM) (*n* = 621). The following patient information was extracted from the registered trauma database: sex; age; trauma mechanisms such as penetrating or blunt injury; comorbidities, which included coronary artery disease (CAD), congestive heart failure (CHF), cerebral vascular accident (CVA), and end-stage renal disease (ESRD); Glasgow Coma Scale (GCS); hypertension (HTN); serum glucose level at the ED; level of hemoglobin A1c; Injury Severity Score (ISS); hospital length of stay (LOS); and hospital mortality.

### 2.3. Methods of Statistical Analysis

We used Statistical Package for the Social Sciences Statistics (SPSS) for Windows, version 23.0 (International Business Machines Corporation, Armonk, NY, USA) for the statistical analysis. The categorical data were analyzed using Pearson chi-squared tests or a two-sided Fisher’s exact test with the presentation of odds ratios (ORs) and 95% confidence intervals (CIs). We used Levene’s test to test the homogeneity of the variance of continuous data. Subsequently, we used a one-way analysis of variance (ANOVA) with the Games–Howell post hoc test to assess the differences of continuous variables among different groups of patients. The continuous data were presented as mean ± standard deviation. The value of ISS was expressed as the median with an interquartile range (Q1–Q3); *p*-values <0.05 were considered statistically significant. The primary outcome of the study was in-hospital mortality. To attenuate the confounding effects of sex, age, comorbidities, and ISS of patients in assessing the mortality rate, different 1:1 propensity score-matched patient populations were established by the NCSS 10 software (NCSS Statistical Software, Kaysville, UT, USA) using the Greedy method in a 0.2 caliper width for the assessment of the impact of admission hyperglycemia (SIH or DH) vs. NDN, as well as SIH vs. DH, on the mortality outcomes.

## 3. Results

### 3.1. Characteristics of the Patients

The characteristics of patients regarding the injuries and outcomes are shown in Table 1. Patient characteristics including age, comorbidities such as preexisting HTN and CHF, and ISS were significantly different among these three groups of patients (SIH, DH, and NDN). On the contrary, sex; comorbidities of CAD, CVA, and ESRD; trauma mechanisms; and GCS among these three groups of patients were insignificantly different. Compared to NDN, DH was observed significantly more in elderly patients (61.4 ± 13.7 vs. 49.8 ± 17.2 years, *p* < 0.001) and in patients with higher incidences of preexisting HTN (2.5% vs. 0.3%, *p* < 0.001) and CHF (3.8% vs. 1.9%, *p* = 0.014). On the contrary, SIH was insignificantly observed in elderly patients and patients with higher incidences of preexisting HTN and CHF, but SIH had a higher ISS (median [Q1–Q3], 20 [15,16,17,18,19,20,21,22] 13 [10,11,12,13,14,15,16,17,18]; *p* < 0.001) than DH, with fewer patients with SIH than with NDN having an ISS <16, and several patients having an ISS of 16 to 24 and ≥25. However, the ISS between DH and NDN was insignificantly different. Regarding patient outcomes, patients with SIH and DH presented 7.2-fold (95% CI, 2.33–22.46; *p* = 0.003) and 4.6-fold (95% CI, 1.50–14.08; *p* = 0.004) higher odds of mortality, respectively, than those with NDN. The hospital LOS was longer in patients with SIH, but not with DH, than in patients with DN (17.4 days vs. 13.1 days, respectively; *p* = 0.014).

### 3.2. Mortality Outcome of the Propensity Score-Matched Patient Populations

Three different propensity score-matched patient populations—SIH vs. NDN (Table 2), DH vs. NDN (Table 3), and SIH vs. DH (Table 4)—were established for the analysis of the mortality outcome. The baseline patient characteristics including sex, age, comorbidities, trauma mechanisms, and ISS were insignificantly different in these established matched patient populations. Patients with SIH and DH had a significantly higher mortality rate than patients with NDN (10.6% vs. 0.0%, *p* = 0.022, and 5.3% vs. 0.0%, *p* = 0.043, respectively). On the contrary, patients with SIH did not present a significantly different mortality rate than patients with DH (11.4% vs. 8.6%; OR, 1.4; 95% CI, 0.29–6.66; *p* = 0.690).

## 4. Discussion

This study revealed that thoracoabdominal injury patients with either SIH or DH had a higher mortality rate than patients with NDN, with or without adjusting the potential confounders such as age, sex, preexisting comorbidities, and injury severity. However, the mortality rate of patients with SIH and DH was significantly different in matched patient populations. This result was in contrary to the study in the general trauma population by Kerby et al. [29] and our study [30], demonstrating that compared to patients with NDN, the adjusted mortality rate was significant higher in patients with SIH, but not in patients with DH. Moreover, this result contradicts our previous report that in the patients with isolated moderate-to-severe traumatic brain injury, there was a significantly higher adjusted mortality rate in the propensity score-matched patients with SIH than the patients with DH [31].

The reason why there were higher odds of mortality in DH patients with a thoracoabdominal injury, but not in those patients with a traumatic brain injury, when compared with the patients with NDN was unknown and thus requires further investigation. Evidently, the pathophysiological response of DH is different from that of SIH. DH is a chronic illness associated with prolonged exposure to hyperglycemia and subsequent microvascular damages [24], while SIH is an acute response that is initiated by stress hormones and the subsequent release of inflammatory cytokines. Therefore, diabetic patients are commonly at higher risk of vascular accidents, such as peripheral vascular disease, cardiovascular problem, cerebrovascular accidents, and renal insufficiency, than patients without diabetes [37,38,39,40,41]. However, little is known whether these vascular accidents have similar effects on mortality in patients with DH with thoracoabdominal injuries or traumatic brain injuries. Additionally, the reasons for the differences in the survival rates and times to mortality of patients with chest trauma vary widely [42]. In a review of 1661 patients with blunt thoracoabdominal injuries, 6.3% of patients had a blunt cardiac trauma and 4.6% had a major thoracic vessel injury [10]. The patients with major trauma to the heart or thoracic vessel may immediately die regardless of their diabetic condition or glucose level. On the contrary, injures to the majority of the solid organs (kidney, 91.2%; liver, 83.9%; and spleen, 68.3%) were managed nonoperatively, and there were rare conditions that required a nonresuscitative thoracotomy or a combined thoracoabdominal operation [10]. A diabetes diagnosis or glucose levels may be more important in determining the mortality outcome for such patients. The results of this study imply that among the patients with different types of admission hyperglycemia, the risk of mortality may depend upon the trauma mechanisms which are associated with immediate or delayed mortality of the patients. However, in this study, considering that the mortality rate of the patients was relatively low and the significant p-value was more marginal (p = 0.043) in patients with DH than in patients with NDN, the unknown effect of etiology on mortality rate in the presence or absence of high glucose levels may result in a bias in the outcome measure, albeit the injury severity being controlled for comparison. Therefore, with additional information about the cause of mortality, a further prospective study of more enrolled patients by different trauma mechanisms would be beneficial to evaluate the effect of SIH and DH on the risk of mortality of the patients.

This study had some other limitations. First, Scalea et al. [43] showed that having a blood glucose level of 100 to 150 mg/dL in critically ill trauma patients significantly reduced mortality rate, while some studies had revealed that a stricter glucose control protocol did not lower the mortality rate of the trauma patients with DH [44,45]. Furthermore, in a prospective, randomized controlled study (NICE-SUGAR) implemented in critically ill patients, the mortality rate was even higher under a tight glucose control than those under conventional glucose control [46]. Although the tight glucose control in patients with hyperglycemia has been inconclusive regarding the reduction of morbidity and mortality rates, as there was no established protocol in treating the admission hyperglycemia, we could only assume that there was a similar management and treatment of these patients. Accordingly, we could only assume that the surgical outcome of patients requiring a thoracotomy or laparotomy by different surgeons is similar. Thirdly, the selection bias with potential unrecognized confounding factors in the retrospective study may be observed. Fourthly, considering that patients declared dead at the scene of an accident or upon arrival to the hospital were not included in the registered trauma database, a selection bias may exist in assessing the mortality outcome.

## 5. Conclusions

This study revealed that thoracoabdominal injury patients with admission hyperglycemia, either SIH or DH, had higher mortality rates than patients with NDN, in the presence or absence of controlling the baseline differences in patients’ age, sex, comorbidities, and injury severity. This result contradicts our previous report that SIH, but not DH, had higher odds of mortality in the patients with isolated moderate-to-severe traumatic brain injuries. The results of this study also implied that in trauma patients with different types of admission hyperglycemia, the risk of mortality may depend on the trauma mechanisms which are associated with different causes of mortality.

## Figures and Tables

**Table 1:** Characteristics and outcomes of patients with stress-induced hyperglycemia, diabetic hyperglycemia, and nondiabetic normoglycemia.

Variables	SIH *n* = 52	DH *n* = 79	NDN *n* = 621	*p*
Male, *n* (%)	35(67.3)	55(69.6)	445(71.7)	0.763
Age, years	50.2 ± 15.6	61.4 ± 13.7 *	49.8 ± 17.2	<0.001
Co-morbidities				
HTN, *n* (%)	10(19.2)	40(50.6) *	112(18.0)	<0.001
CHF, *n* (%)	0(0.0)	2(2.5) *	2(0.3)	0.034
CAD, *n* (%)	1(1.9)	3(3.8)	12(1.9)	0.555
CVA, *n* (%)	1(1.9)	1(1.3)	7(1.1)	0.878
ESRD, *n* (%)	0(0.0)	2(2.5)	9(1.4)	0.498
Mechanisms				0.642
Penetrating injury, *n* (%)	2(3.8)	1(1.3)	17(2.7)	
Blunt injury, *n* (%)	50(96.2)	78(98.7)	604(97.3)	
GCS	14.0 ± 1.9	14.2 ± 2.6	14.5 ± 1.8	0.167
ISS, median (IQR)	20(15–22) *	13(9–17)	13(10–18)	<0.001
<16	13(25.0) *	48(60.8)	356(57.3)	<0.001
16–24	30(57.7) *	23(29.1)	223(35.9)	0.002
≥25	9(17.3) *	8(10.1)	42(6.8)	0.018
Mortality, *n* (%)	5(9.6) *	5(6.3) *	9(1.4)	<0.001
Hospital LOS (days)	17.4 ± 17.7 *	14.7 ± 14.5	13.1 ± 10.7	0.036

HTN = hypertension; CHF = congestive heart failure; CAD = coronary artery disease; CVA = cerebral vascular accident; GCS = Glasgow Coma Scale; ISS = injury severity score; IQR = interquartile range; LOS = length of stay; SIH = stress-induced hyperglycemia; DH = diabetic hyperglycemia; NDN = nondiabetic normoglycemia; ESRD = end-stage renal disease. * indicate p < 0.05 when compare NDN.

**Table 2:** The mortality outcome in the matched patient populations with stress-induced hyperglycemia and nondiabetic normoglycemia.

	Propensity-Score Matched Cohort				
Variables	SIH *n* = 47	NDN *n* = 47	Odds Ratio (95% CI)	*p*	Standardized Difference
Male, *n* (%)	14 (29.8)	14 (29.8)	1.0 (0.41–2.42)	1.000	0.00%
Age, years	50.2 ± 15.5	50.3 ± 15.5	―	0.963	−0.96%
Co-morbidities					
HTN, *n* (%)	9 (19.1)	9 (19.1)	1.0 (0.36–2.79)	1.000	0.00%
CAD, *n* (%)	0 (0.0)	0 (0.0)	―	―	―
CHF, *n* (%)	0 (0.0)	0 (0.0)	―	―	―
CVA, *n* (%)	0 (0.0)	0 (0.0)	―	―	―
ESRD, *n* (%)	0 (0.0)	0 (0.0)	―	―	―
Mechanisms, *n* (%)					0.00%
Penetrating injury, *n* (%)	1 (2.1)	1 (2.1)	1.0 (0.06–16.47)	1.000	
Blunt injury, *n* (%)	46 (97.9)	46 (97.9)	1.0 (0.06–16.47)	1.000	
ISS, median (IQR)	20 (14–21)	19 (14–21)	―	0.985	−0.38%
Outcome measurement					
Mortality, *n* (%)	5 (10.6)	0 (0.0)	―	0.022	―

HTN = hypertension; CHF = congestive heart failure; CAD = coronary artery disease; CVA = cerebral vascular accident; ISS = injury severity score; IQR = interquartile range; SIH = stress-induced hyperglycemia; NDN = nondiabetic normoglycemia.

**Table 3:** The mortality outcome in the matched patient populations with diabetic hyperglycemia and nondiabetic normoglycemia.

	Propensity-Score Matched Cohort				
Variables	DH *n* = 76	NDN *n* = 76	Odds Ratio (95% CI)	*p*	Standardized Difference
Male, *n* (%)	53 (69.7)	53 (69.7)	1.0 (0.50–2.00)	1.000	0.00%
Age, years	61.1 ± 13.8	61.2 ± 13.1	―	0.986	−0.29%
Co-morbidities					
HTN, *n* (%)	37 (48.7)	37 (48.7)	1.0 (0.53–1.89)	1.000	0.00%
CAD, *n* (%)	1 (1.3)	1 (1.3)	1.0 (0.06–16.29)	1.000	0.00%
CHF, *n* (%)	0 (0.0)	0 (0.0)	―	―	―
CVA, *n* (%)	1 (1.3)	1 (1.3)	1.0 (0.06–16.29)	1.000	0.00%
ESRD, *n* (%)	1 (1.3)	1 (1.3)	1.0 (0.06–16.29)	1.000	0.00%
Mechanisms, *n* (%)					0.00%
Penetrating injury, *n* (%)	1 (1.3)	1 (1.3)	1.0 (0.06–16.29)		
Blunt injury, *n* (%)	75 (98.7)	75 (98.7)	1.0 (0.06–16.29)		
ISS, median (IQR)	13 (9–17)	14 (9–17)	―	0.987	−0.46%
Outcome measurement					
Mortality, *n* (%)	4 (5.3)	0 (0.0)	―	0.043	―

HTN = hypertension; CHF = congestive heart failure; CAD = coronary artery disease; CVA = cerebral vascular accident; ISS = injury severity score; IQR = interquartile range; DH = diabetic hyperglycemia; NDN = nondiabetic normoglycemia.

**Table 4:** The mortality outcome in the matched patient populations with stress-induced hyperglycemia and diabetic hyperglycemia.

	Propensity-Score Matched Cohort				
Variables	SIH *n* = 35	DH *n* = 35	Odds Ratio (95% CI)	*p*	Standardized Difference
Male, *n* (%)	24 (68.6)	24 (68.6)	1.0 (0.36–2.74)	1.000	0.00%
Age, years	54.6 ± 14.6	55.1 ± 13.9	―	0.881	−3.60%
Co-morbidities					
HTN, *n* (%)	9 (25.7)	9 (25.7)	1.0 (0.34–2.92)	1.000	0.00%
CAD, *n* (%)	0 (0.0)	0 (0.0)	―	―	―
CHF, *n* (%)	0 (0.0)	0 (0.0)	―	―	―
CVA, *n* (%)	0 (0.0)	0 (0.0)	―	―	―
ESRD, *n* (%)	0 (0.0)	0 (0.0)	―	―	―
Mechanisms, *n* (%)					0.00%
Penetrating injury, *n* (%)	0 (0.0)	0 (0.0)	―	―	―
Blunt injury, *n* (%)	35 (100)	35 (100)	―	―	―
ISS, median (IQR)	17 (13–20)	17 (10–21)	―	0.706	9.06%
Outcome measurement					
Mortality, *n* (%)	4 (11.4)	3 (8.6)	1.4 (0.29–6.66)	0.690	―

HTN = hypertension; CHF = congestive heart failure; CAD = coronary artery disease; CVA = cerebral vascular accident; ISS = injury severity score; IQR = interquartile range; SIH = stress-induced hyperglycemia; DH = diabetic hyperglycemia.
